# Prospecting for Zoonotic Pathogens by Using Targeted DNA Enrichment

**DOI:** 10.3201/eid2908.221818

**Published:** 2023-08

**Authors:** Egie E. Enabulele, Winka Le Clec’h, Emma K. Roberts, Cody W. Thompson, Molly M. McDonough, Adam W. Ferguson, Robert D. Bradley, Timothy J. C. Anderson, Roy N. Platt

**Affiliations:** Texas Biomedical Research Institute, San Antonio, Texas, USA (E.E. Enabulele, W. Le Clec’h, T.J.C. Anderson, R.N. Platt II);; Texas Tech University, Lubbock, Texas, USA (E.K. Roberts, R.D. Bradley);; University of Michigan, Ann Arbor, Michigan, USA (C.W. Thompson);; Chicago State University, Chicago, Illinois, USA (M.M. McDonough);; Field Museum of Natural History, Chicago (A.W. Ferguson)

**Keywords:** prospecting, zoonotic pathogens, targeted DNA enrichment, bacteria, genetic archive, natural history museum, zoonoses, United States

## Abstract

More than 60 zoonoses are linked to small mammals, including some of the most devastating pathogens in human history. Millions of museum-archived tissues are available to understand natural history of those pathogens. Our goal was to maximize the value of museum collections for pathogen-based research by using targeted sequence capture. We generated a probe panel that includes 39,916 80-bp RNA probes targeting 32 pathogen groups, including bacteria, helminths, fungi, and protozoans. Laboratory-generated, mock-control samples showed that we are capable of enriching targeted loci from pathogen DNA 2,882‒6,746-fold. We identified bacterial species in museum-archived samples, including *Bartonella*, a known human zoonosis. These results showed that probe-based enrichment of pathogens is a highly customizable and efficient method for identifying pathogens from museum-archived tissues.

Many serious human pathogens result from zoonotic transmission, including 61% of known human pathogens and 75% of emerging human pathogens (*1*). For example, rabies virus is transmitted by saliva of infected animals (*2*). The plague bacteria (*Yersina pestis*), the causative agent of the largest documented pandemic in human history that reduced the population of Europe by 30%–50%, was transmitted from rats to humans by fleas (*3*). Other zoonoses include Ebola virus (*4*), tularemia (*Francisella tularensis*) (*5*), and tuberculosis (*6*). The SARS-CoV-2 pandemic, thought to have a bat reservoir, has stimulated renewed emphasis on zoonotic pathogen surveillance (*7*,*8*).

Natural history museums are repositories of biologic information in the form of voucher specimens that represent a major, underused resource for studying zoonotic pathogens (*9*–*13*). Originally, specimens were archived as dried skin and skeletal vouchers or preserved in fluids (ethanol) after fixation with formalin or formaldehyde. Now, best practices include preserving specimens and associated soft tissues in liquid nitrogen (−190°C) or mechanical freezers (−80°C) from the time they are collected (*14*). Those advances in preservation make it possible to extract high-quality DNA and RNA that can be used for pathogen surveillance. For example, retroactive sampling of archived tissues from the US Southwest found that Sin Nombre virus, a New World hantavirus, was circulating in wild rodent populations almost 20 years before the first human cases were reported (*15*).

It is critical to develop a range of tools for extracting pathogen information from museum-archived samples. Targeted sequencing using probe enrichment has become the tool of choice for medical genomics (*16*), population genetics (*17*), phylogenetics (*18*), and ancient DNA (*19*,*20*). Those methods are designed to enrich small amounts of DNA target from a background of contaminating DNA. Probe-based, targeted sequencing has been used to enrich pathogens from complex host‒pathogen DNA mixtures (*21*). For example, Keller et al. used probes to capture and sequence complete *Y. pestis* genomes from burial sites >1,500 years old (*22*). Enrichment is frequently achieved by designing a panel of probes to specifically target a handful of pathogens of interest (*23*,*24*). Similarly, commercial probe sets are available for many types of viruses and human pathogens (*23*–*25*). However, many of these probe sets are limited to specific pathogens that might not infect other host species.

Our goal was to develop a panel of biotinylated baits, or probes, to identify the eukaryotic and bacterial pathogens responsible for 32 major zoonoses (Table 1). We aimed to capture both known and related pathogens, using the fact that probes can capture sequences that are ≤10% divergent. To perform this capture, we used a modified version of the ultraconserved element (UCE) targeted sequencing technique (*26*,*27*) to specifically enrich pathogen DNA. Biotinylated baits are designed to target conserved genomic regions among diverse groups of pathogens (Figure 1). The baits are hybridized to a library potentially containing pathogen DNA. Bait-bound DNA fragments are enriched during a magnetic bead purification step before sequencing (Figure 2). The final library contains hundreds or thousands of orthologous loci with single-nucleotide variants or indels from the targeted pathogen groups that can then be used for population or phylogenetic analyses.

**Table 1:** Zoonotic pathogens targeted for DNA enrichment in study of prospecting for zoonotic pathogens by using targeted DNA enrichment

Pathogen group	Taxonomic level	Focal pathogen	Zoonoses
*Anaplasma*	Genus	*Anaplasma phagocytophilum*	Anaplasmosis
Apicomplexa	Phylum	*Plasmodium falciparum*	Malaria
*Bacillus cereus* group*	Species group	*Bacillus anthracis*	Anthrax
*Bartonella*	Genus	*Bartonella bacilliformis*	Cat-scratch fever
*Borrelia*	Genus	*Borrelia burgdorferi*	Lyme disease
*Burkholderia*	Genus	*Burkholderia mallei*	Glanders
*Campylobacter*	Genus	*Campylobacter jejuni*	Campylobacteriosis
Cestoda	Class	*Taenia multiceps*	Taeniasis
*Chlamydia*	Genus	*Chlamydia trachomatis*	Chlamydia
*Coxiella*	Genus	*Coxiella burnetii*	Q fever
*Ehrlichia*	Genus	*Ehrlichia canis*	Ehrlichiosis
Eurotiales	Order	*Talaromyces marneffei*	Talaromycosis
*Francisella*	Genus	*Francisella tularensis*	Tularemia
Hexamitidae	Family	*Giardia intestinalis*	Giardiasis
Kinetoplastea	Class	*Leishmania major*	Leishmaniasis
*Leptospira*	Genus	*Leptospira interrogans*	Leptospirosis
*Listeria*	Genus	*Listeria monocytogenes*	Listeriaosis
*Mycobacterium*	Genus	*Mycobacterium tuberculosis*	Tuberculosis
Nematodes (clade I)	Phylum (clade)	*Trichinella spiralis*	Trichinosis
Nematodes (clade III)	Phylum (clade)	*Brugia malayi*	Filariasis
Nematodes (clade IVa)	Phylum (clade)	*Strongyloides stercoralis*	Strongyloidiasis
Nematodes (clade IVb)	Phylum (clade)	*Steinernema carpocapsae*	None
Nematodes (clade V)	Phylum (clade)	*Haemonchus contortus*	None
Onygenales	Order	*Histoplasma capsulatum*	Histoplasmosis
*Pasteurella*	Genus	*Pasteurella multocida*	Pasteurellosis
*Rickettsia*	Genus	*Rickettsia rickettsii*	Typhus
*Salmonella*	Genus	*Salmonella enterica*	Salmonellosis
*Streptobacillus*	Genus	*Streptobacillus moniliformis*	Rat-bite fever
Trematoda	Class	*Schistosoma mansoni*	Schistosomiasis
Tremellales	Order	*Cryptococcus neoformans*	Cryptococcosis
Trypanosoma*	Genus	*Trypanosoma cruzi*	Sleeping sickness
*Yersinia*	Genus	*Yersinia pestis*	Plague

*Supplemented with additional probes/baits.

**Figure 1:**
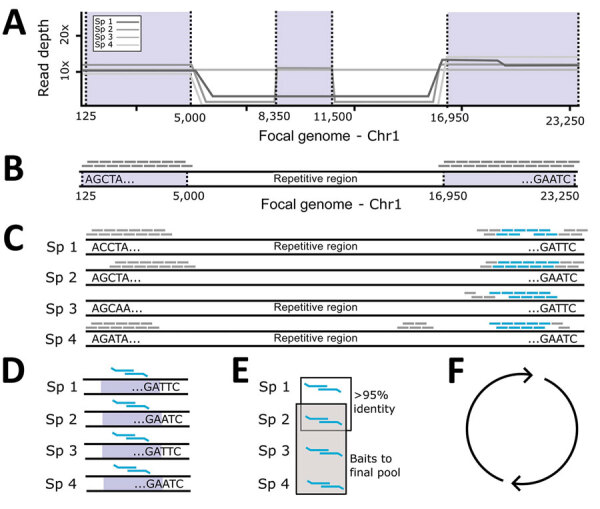
Probe panel design for study of prospecting for zoonotic pathogens by using targeted DNA enrichment. A) Simulated reads from each pathogen within a group were mapped back to a single focal genome. B) We identified regions with consistent coverage from each member of the pathogen group to identify putative, orthologous loci and generated a set of in silico probes from the focal genome. C) Those in silico probes were then mapped back to the genomes of each member in the pathogen group to find single copy, orthologous regions, present in most members. D, E) We designed 2 overlapping 80-bp baits to target the loci in each member of the pathogen group (D) and compared them with each another to remove highly similar probes (E). One probe was retained from each group of probes with high sequence similarity (>95%). F) We identified the probes necessary to capture 49 loci in that pathogen group. This process was repeated for the next pathogen group. Finally, all probes were combined together into a single panel. Chr, chromosome; Sp, specimen.

**Figure 2:**
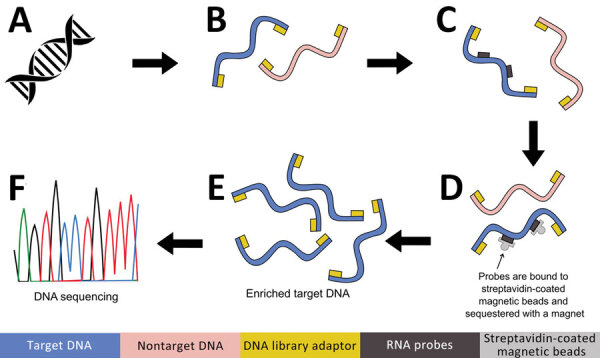
Targeted DNA enrichment workflow for study of prospecting for zoonotic pathogens by using targeted DNA enrichment. A) Genomic DNA extracted using the DNeasy Kit (QIAGEN, https://www.qiagen.com). B) Next-generation sequencing libraries prepared using KAPA Hyperplus Kit (https://www.biocompare.com) and barcoding each library with IDT xGen Stubby Adaptor-UDI Primers (https://www.idtdna.com). C) RNA probes hybridization using the high sensitivity protocol of myBaits version 5. (https://arborbiosci.com). D) Probes bound to streptavidin-coated magnetic beads and sequestered with a magnet (E) 15 cycles PCR amplification of enriched libraries. F) Libraries sequenced on an Illumina Hi-Seq 2500 platform (https://www.illumina.com).

## Methods

We have compiled a detailed description of the methods used (Appendix 1; https://doi.org/10.17504/protocols.io.5jyl8jnzrg2w/v1). Code is available on GitHub (https://www.github.com/nealplatt/pathogen_probes; https://doi.org/10.5281/zenodo.7319915). Raw sequence data are available from the National Center for Biotechnology Information (BioProject PRJNA901509; Appendix 2). A summary of our methods follows.

### Panel Development

We developed a panel of baits for targeted sequencing of 32 zoonotic pathogens. To develop this panel, we used the Phyluce version 1.7.1 (*26*,*27*) protocol to design baits for conserved loci within each pathogen group. First, we simulated and mapped reads from each species within a pathogen group to a focal genome assembly (Table 1; Figure 1, panel A). We used the mapped reads to identify putative orthologous loci that were >80% similar across the group and generated in silico baits from the focal genome (Figure 1, panel B). These baits were mapped back to each member (Figure 1, panel C) to identify single-copy orthologs within the group. Next, we designed 2 overlapping 80-bp baits from loci in each member of the group (Figure 1, panel D) and removed baits with >95% sequence similarity (Figure 1, panel E). We repeated those steps for each pathogen group (Figure 1, panel F). We compared the remaining baits with mammalian genomes and replaced them to minimize cross-reactivity with the host. Finally, we combined baits to capture 49 loci from each pathogen group into a panel that was synthesized by Daicel Arbor Biosciences (https://arborbiosci.com).

### Museum-Archived and Control Samples

We extracted DNA from 38 museum samples by using the DNeasy Kit (QIAGEN, https://www.qiagen.com) (Table 2). We generated control samples by spiking naive mouse DNA with 1% microorgamism DNA from *Mycobacterium bovis*, *M. tuberculosis*, *Plasmodium vivax*, *P. falciparum*, and *Schistosoma mansoni.* We then further diluted an aliquot of this 1% pathogen mixture into mouse DNA to create a 0.001% host‒pathogen mixture. This range was designed to test the lower limits of detection but also represent a reasonable host‒pathogen proportion. For example, *Theileria parva*, a tick-transmitted apicomplexan, is present in samples from 0.9% through 3% (*28*), and 1.5% of DNA sequence reads in clinical blood samples is from *P. vivax* (*29*).

**Table 2:** Specimens examined using targeted sequencing in study of prospecting for zoonotic pathogens by using targeted DNA enrichment*

Museum accession no.	Source species (common name)	Locality, country: state, county	Date	SRA ID
TK48533	*Myotis volans* (long-legged myotis)	Mexico: Durango, Arroyo El Triguero	1995 May 18	SAMN31718202
TK49668	*Didelphis virginiana *(Virginia opossum)	United States: Texas, Kerr	1996 May 14	SAMN31718203
TK49674	*Peromyscus attwateri *(Texas mouse)	United States: Texas, Kerr	1996 May 14	SAMN31718204
TK49686	*Peromyscus laceianus *(deer mouse)	United States: Texas, Kerr	1996 May 14	SAMN31718205
TK49712	*Dasypus novemcinctus *(nine-banded armadillo)	United States: Texas, Kerr	1996 May 16	SAMN31718206
TK49732	*Lasiurus borealis *(eastern red bat)	United States: Texas, Kerr	1996 May 17	SAMN31718207
TK49733	*Myotis velifer *(vesper bat)	United States: Texas, Kerr	1996 May 16	SAMN31718208
TK57832	*P. attwateri*	United States: Texas, Kerr	1997 May 14	SAMN31718209
TK70836	*Desmodus rotundus *(common vampire bat)	Mexico: Durango, San Juan de Camarones	1997 Jun 27	SAMN31718210
TK90542	*Sigmodon hirsutus *(southern cotton rat)	Mexico: Chiapas, Comitán	1999 Jul 9	SAMN31718211
TK93223	*Peromyscus melanophrys *(plateau mouse)	Mexico: Oaxaca, Las Minas	2000 Jul 13	SAMN31718212
TK93289	*Carollia subrufa *(gray short-tailed bat)	Mexico: Chiapas, Ocozocoautla	2000 Jul 16	SAMN31718213
TK93402	*Chaetodipus eremicus *(Chihuahan pocket mouse)	Mexico: Coahuila	2000 Jul 22	SAMN31718214
TK101275	*Glossophaga commissarisi *(Commissaris’ long-tongued bat)	Honduras: Comayagua, Playitas	2001 Jul 10	SAMN31718215
TK136205	*Heteromys desmarestianus *(Desrmarest’s spiny pocket mouse)	Honduras: Atlantida, Jardin Botanico Lancetilla	2004 Jul 16	SAMN31718216
TK136222	*Peromyscus mexicanus *(Mexican deer mouse)	Honduras: Colon, Trujillo	2004 Jul 17	SAMN31718217
TK136228	*H. desmarestianus*	Honduras: Colon, Trujillo	2004 Jul 17	SAMN31718218
TK136240	*Glossophaga soricine *(Pallas’s long-tongued bat)	Honduras: Colon, Trujillo	2004 Jul 16	SAMN31718219
TK136756	*Eptesicus furinalis *(Argentine brown bat)	Honduras: Colon, Trujillo	2004 Jul 17	SAMN31718220
TK136783	*Glossophaga leachii *(gray long-tongued bat)	Honduras: Colon, Trujillo	2004 Jul 17	SAMN31718221
TK148935	*Rhogeessa tumida *(back-winged little yellow bat)	Mexico: Tamaulipas, Soto la Marina	2008 Jul 27	SAMN31718222
TK148943	*M. velifer*	Mexico: Tamaulipas, Soto la Marina	2008 Jul 27	SAMN31718223
TK150290	*Balantiopteryx plicata *(gray sac-winged bat)	Mexico: Michoacan, El Marqués	2006 Jul 22	SAMN31718224
TK154677	*Gerbilliscus leucogaster *(bushveld gerbil)	Botswana: Ngamiland, Koanaka Hills	2008 Jun 29	SAMN31718225
TK154685	*G. leucogaster*	Botswana: Ngamiland, Koanaka Hills	2008 Jun 29	SAMN31718226
TK154687	*G. leucogaster*	Botswana: Ngamiland, Koanaka Hills	2008 Jun 29	SAMN31718227
TK164683	*Mastomys natalensis *(Natal multimammate mouse)	Botswana: Ngamiland, Koanaka Hills	2009 Jul 18	SAMN31718228
TK164686	*M. natalensis*	Botswana: Ngamiland, Koanaka Hills	2009 Jul 18	SAMN31718229
TK164689	*M. natalensis*	Botswana: Ngamiland, Koanaka Hills	2009 Jul 18	SAMN31718230
TK164690	*M. natalensis*	Botswana: Ngamiland, Koanaka Hills	2009 Jul 18	SAMN31718231
TK164702	*M. natalensis*	Botswana: Ngamiland, Koanaka Hills	2009 Jul 19	SAMN31718232
TK164714	*M. natalensis*	Botswana: Ngamiland, Koanaka Hills	2009 Jul 19	SAMN31718233
TK164728	*M. natalensis*	Botswana: Ngamiland, Koanaka Hills	2009 Jul 19	SAMN31718234
TK166246	*P. attwateri*	United States: Texas, Kerr	2010 May 17	SAMN31718235
TK179690	*P. attwateri*	United States: Texas, Kerr	2013 May 20	SAMN31718236
TK185677	*P. attwateri*	United States: Texas, Kerr	2018 May 21	SAMN31718237
TK197046	*P. attwateri*	United States: Texas, Kerr	2016 May 26	SAMN31718238
TK199855	*P. attwateri*	United States: Texas, Kerr	2019 May 21	SAMN31718239

*ID, identification; SRA, National Center for Biotechnology Information Sequence Read Archive.

### Library Preparation

We generated standard DNA sequencing libraries from 500 ng of DNA per sample. We combined individual libraries with similar DNA concentrations into pools of 4 samples and used the myBaits version 5 (Daicel Arbor Biosciences) high sensitivity protocol to enrich target loci. We used 2 rounds of enrichment (24 h at 65°C), washed away unbound DNA, and amplified the remainder for 15 cycles before pooling for sequencing.

### Classifying Reads

**Figure 3:**
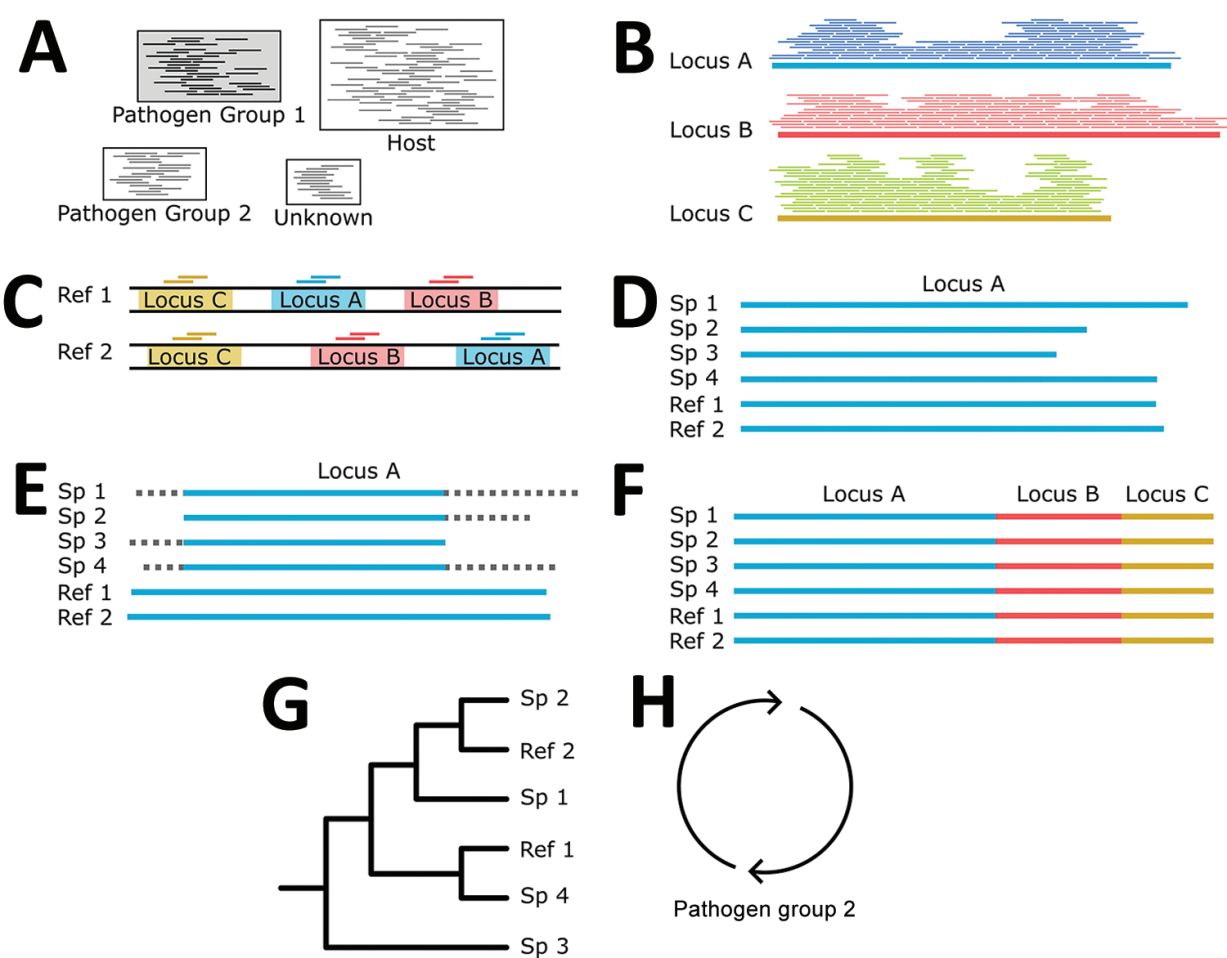
Building phylogenies from parasite reads for study of prospecting for zoonotic pathogens by using targeted DNA enrichment. A) After read classification, we extracted all the reads associated with a pathogen group. B) Those reads were assembled into contigs with a genome assembler. C) Simultaneously, we identified and extracted the target loci from all members of the pathogen group with available reference genomes to ensure that our final phylogeny has representatives from as many members of the pathogen group as possible. D, E) For each targeted locus, we combined the assembled contigs (D) and genome extracted loci for (E) multiple sequence alignment and trimming. F, G) Each aligned and trimmed locus is concatenated together (F) for phylogenetic analyses (G). H) If necessary, those steps are repeated for reads classified in other pathogen groups. Ref, reference; Sp, specimen.

First, we generated a dataset of target loci by mapping the probes to representative and reference genomes in RefSeq v212 with BBMap v38.96 (*30*). For each probe, we kept the 10 best sites that mapped with >85% sequence identity along with 1,000 bp upstream and downstream. These sequences were combined into a database to classify reads by using Kraken2 version 2.1.1 (*31*) (Figure 3, panel A). Next, we extracted pathogen reads with KrakenTools version 1.2 (https://github.com/jenniferlu717/KrakenTools). We assembled those reads (Figure 3, panel B) with the SPAdes genome assembler version 3.14.1 (*32*) and filtered them to remove low quality contigs (<100 bp and <10× median coverage). We removed samples that had <2 contigs from downstream analyses. During this time, we extracted target loci in available reference genomes (Figure 3, panel C). Next, we identified (Figure 3, panel D), aligned and trimmed (Figure 3, panel E) orthologs before concatenating them into a single alignment (Figure 3, panel F). Finally, we generated and bootstrapped a phylogenetic tree (Figure 3, panel G) by using RaxML-NG version 1.0.1 (*33*). We repeated those steps for each pathogen group (Figure 3, panel H).

### Host Identification

There were sufficient mtDNA sequences from most samples to verify museum identifications by comparing reads to a Kraken2 version 2.1.2 (*31*) database of mammalian mitochondrial genomes. We filtered the classifications by removing samples with <50 classified reads and single-read, generic classifications.

## Results

### Panel Development

We used the ultraconserved element protocol developed by Faircloth et al. (*26*,*27*) to develop a set of 39,893 biotinylated baits that target 32 pathogen groups responsible for 32 zoonoses. Each pathogen group is targeted at 49 loci with a few diverse taxa, *Bacillus cereus* and *Trypanosoma* species, targeted at 98 loci. We complied information on pathogen groups, focal taxa, genome accessions, and number of baits (Table 3).

**Table 3:** Summary of probes developed for targeted capture of pathogen DNA in study of prospecting for zoonotic pathogens by using targeted DNA enrichment

Pathogen group	Type	Probe count	Locus count	RefSeq genome count	Focal pathogen	GenBank accession no.
*Anaplasma*	Bacteria	368	49	57	*Anaplasma phagocytophilum*	GCF000013125
Apicomplexa	Eukaryote	3,219	49	64	*Plasmodium falciparum*	GCA000002765
*Bacillus cereus* group*	Bacteria	833	98	134	*Bacillus anthracis*	GCF000008165
*Bartonella*	Bacteria	1,812	49	31	*Bartonella bacilliformis*	GCF000015445
*Borrelia*	Bacteria	688	49	16	*Borreliella burgdorferi*	GCF000502155
*Burkholderia*	Bacteria	683	49	39	*Burkholderia mallei*	GCF000011705
*Campylobacter*	Bacteria	2,194	49	33	*Campylobacter jejuni*	GCF000009085
Cestoda	Eukaryote	907	49	18	*Taenia multiceps*	GCA001923025
*Chlamydia*	Bacteria	830	49	15	*Chlamydia trachomatis*	GCF000008725
*Coxiella*	Bacteria	144	49	70	*Coxiella burnetii*	GCF000007765
*Ehrlichia*	Bacteria	235	49	7	*Ehrlichia canis*	GCF000012565
Eurotiales	Eukaryote	4,097	49	158	*Talaromyces marneffei*	GCF000001985
*Francisella*	Bacteria	470	49	14	*Francisella tularensis*	GCF000008985
Hexamitidae	Eukaryote	782	49	19	*Giardia intestinalis*	GCA000002435
Kinetoplastea	Eukaryote	2,917	49	49	*Leishmania major*	GCF000002725
*Leptospira*	Bacteria	2,517	49	69	*Leptospira interrogans*	GCF000092565
*Listeria*	Bacteria	765	49	23	*Listeria monocytogenes*	GCF000196035
*Mycobacterium*	Bacteria	2,463	49	86	*Mycobacterium tuberculosis*	GCF000195955
Nematodes, clade I	Eukaryote	357	49	13	*Trichinella spiralis*	GCA000181795
Nematodes, clade III	Eukaryote	1,494	49	25	*Brugia malayi*	GCA000002995
Nematodes, clade IVa	Eukaryote	252	49	7	*Strongyloides stercoralis*	GCA000947215
Nematodes, clade IVb	Eukaryote	1,487	43	34	*Steinernema carpocapsae*	GCA000757645
Nematodes, clade V	Eukaryote	3,242	48	47	*Haemonchus contortus*	GCA007637855
Onygenales	Eukaryote	1,973	49	38	*Histoplasma capsulatum*	GCF000149585
*Pasteurella*	Bacteria	615	49	11	*Pasteurella multocida*	GCF000754275
*Rickettsia*	Bacteria	394	49	37	*Rickettsia rickettsii*	GCF001951015
*Salmonella*	Bacteria	145	49	35	*Salmonella enterica*	GCF001159405
*Streptobacillus*	Bacteria	245	49	7	*Streptobacillus moniliformis*	GCF000024565
Trematoda	Eukaryote	924	49	18	*Schistosoma mansoni*	GCA000237925
Tremellales	Eukaryote	1,999	49	26	*Cryptococcus neoformans*	GCF000091045
Trypanosoma*	Eukaryote	617	97	10	*Trypanosoma cruzi*	GCF000209065
*Yersinia*	Bacteria	225	49	22	*Yersinia pestis*	GCF000009065

*Supplemented.

### Control Samples

**Figure 4:**
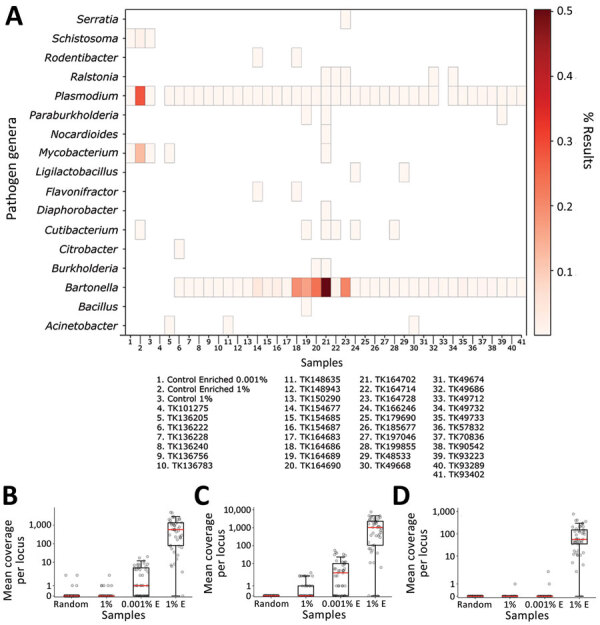
Identifying pathogen reads from controls and museum-archived tissue samples for study of prospecting for zoonotic pathogens by using targeted DNA enrichment. Control reads are indicated by the percentage of pathogen DNA 1% or 0.001%. A) Reads were compared with a database of target loci and assigned a taxonomic classification based on these results. Reads were assigned to 93 genera; of those, 17 (shown) were present in >1 sample, including controls, with ≥1,000 reads. A heatmap of those results shows the relative proportion of reads assigned to each genus. Details of samples are provided in Table 2. B–D) Coverage at each probed locus is shown across all control samples for *Mycobacterium *(B), *Plasmodium *(C), and *Schistosoma *(D). Each point in the chart is coverage calculated at a single target locus. Horizontal lines within boxes indicate medians, box tops and bottoms indicate lower and upper quartiles, and whiskers represent minimum and maximum values, excluding outliers. Each sample is indicated with a circle. E, enriched.

We tested the efficacy of our bait set on laboratory-made host‒pathogen mixtures containing DNA from *Mus musculus*, *Mycobacterium tuberculosis*, *Plasmodium falciparum*, *P. vivax* and *Schistosoma mansoni*. We generated 4 control samples containing either 1% or 0.001% pathogen DNA that was enriched or not enriched. We classified reads against the database of target loci and found that 42.7% of all reads (*Mycobacterium* = 13.1%, *Plasmodium* = 28.1%, *Schistosoma* = 1.5%) were from control pathogens in the 1% enriched control sample. However, only 0.03% of the corresponding 1% unenriched control was from target loci. Aside from the raw percentages, we compared the coverage of each probed region in the 1% enriched and unenriched control samples (Figure 4, panels B‒D) to understand how enrichment effected coverage at each locus. Mean coverage per *Mycobacterium* locus increased from 0.14× to 944.5× (6,746-fold enrichment), 0.53× to 1,527.4× for *Plasmodium* loci (2,882-fold enrichment), and 0.02× to 117.9× (5,895-fold enrichment) for schistosome loci. Because the sequencing library from the 0.001% unenriched sample did not work during the sequencing reaction, we do not have a baseline to examine enrichment in the 0.001% samples.

**Figure 5:**
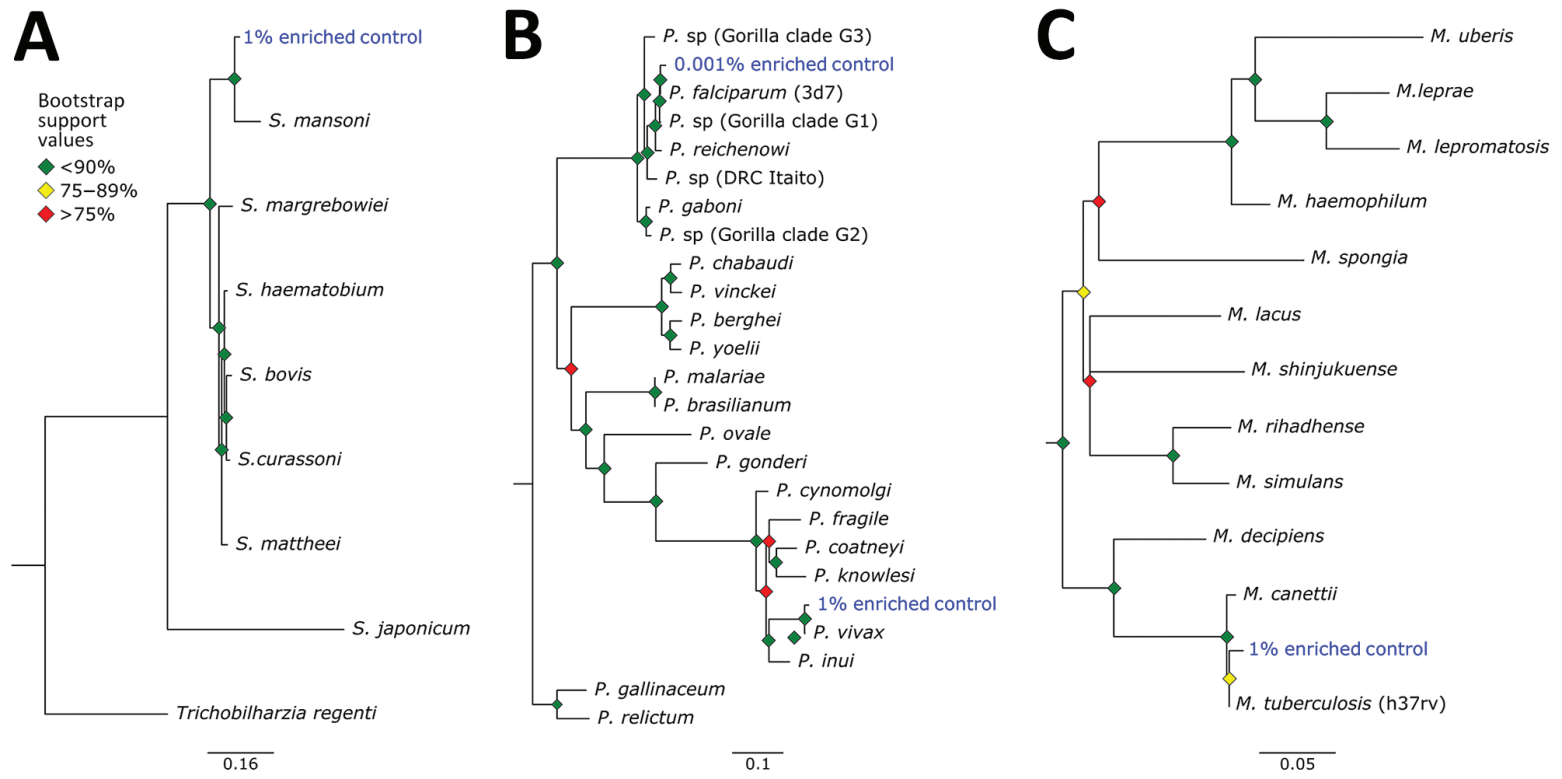
Phylogenetic analysis of pathogens used in control samples for study of prospecting for zoonotic pathogens by using targeted DNA enrichment. A) *Schistosoma*; B) *Plasmodium*; C) *Mycobacterium*. Reads from each control pathogen (*M. tuberculosis*, *P. falciparum*, *P. vivax*, and *S. mansoni*) were extracted, assembled, aligned, and trimmed for maximum-likelihood phylogenetic analyses. The phylogenies were used to identify the species or strain of pathogen used in the controls. Blue indicates control samples. Bootstrap support values are indicated by colored diamonds at each available node. Branches with <50% bootstrap support were collapsed. Nodal support is indicated by color coded diamonds. Scale bars indicate nucleotide substitutions per site. Assembly accession numbers (e.g., GCA902374465) and tree files are available from https://doi.org/10.5281/zenodo.8014941.

We extracted reads assigned to each pathogen group and assembled and aligned them with target loci extracted from reference genomes of closely related species by using tools from Phyluce version 1.7.1 (*26*,*27*). We were able to assemble 0–23 target loci per pathogen group in the control samples (Table 4). Assembled loci varied in size from 109 to 1,991 bp (median 636.5 bp). For each sample/group with >2 loci captured, we generated a phylogenetic tree along with other members of the taxonomic group (Figure 5). In each case, pathogen loci from the control samples were sister groups to the appropriate reference genome with strong bootstrap support. For example, the *Schistosoma* loci assembled from the 1% enriched control sample were sister to the *S.* *mansoni* genome (GCA000237925) in 100% of bootstrap replicates.

**Table 4:** Parasite reads identified in and loci assembled from control samples

Enriched	Pathogen concentration, %	Total reads	*Schistosoma*			*Plasmodium*			*Mycobacterium*	
			Reads	Loci		Reads	Loci		Reads	Loci
True	0.001	509,672	3	0		168	7		556	0
True	1	398,469	5,879	23		52,274	8		112,141	23
False	1	375,786	15	0		17	0		83	0

### Museum Samples

Next, we tested our bait set on museum-archived tissues. We generated 649.3 million reads across all 38 samples (mean 17.1 million reads/sample). An initial classification showed that, on average, 4.3% of reads were assignable to loci in the database. Those reads were designated to 93 genera. However, 78 of those genera were at low frequency (<1,000 reads/sample) (Figure 4). Many of the low frequency hits are likely the result of bioinformatic noise. *Bartonella* and *Plasmodium* species were the most common genera; each was present in 36 of 38 museum samples. The distribution of *Bartonella* reads was strongly bimodal such that 18 samples had <12 reads and 18 samples had >1,000 reads (median 552 reads/sample). In 5 samples, the percentage of *Bartonella* reads was exceedingly high (>10%). In comparison, the median number of *Plasmodium* reads never exceeded 0.04% of reads from a single museum sample (mean 158.5 reads/sample).

**Figure 6:**
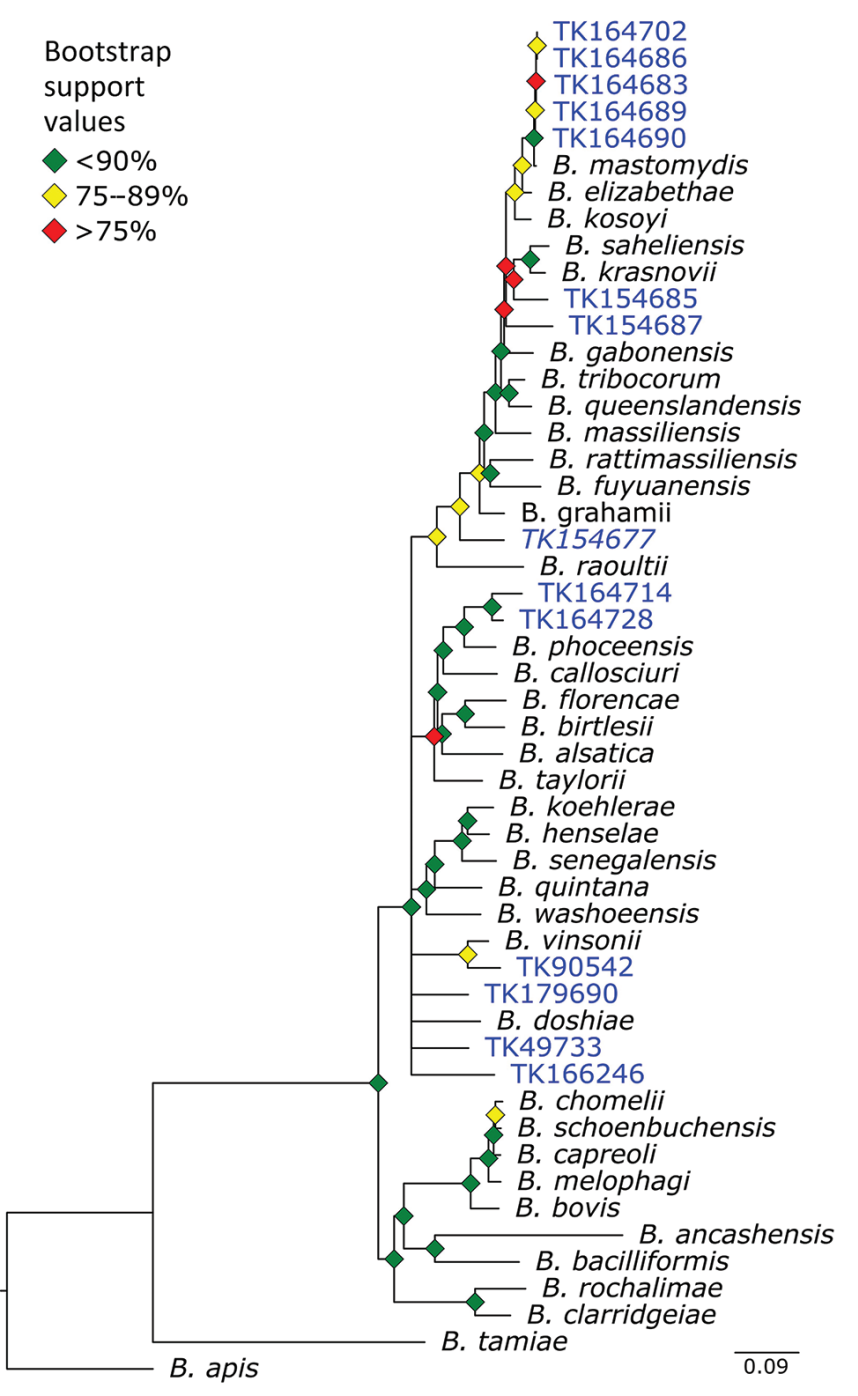
Phylogenetic analysis of *Bartonella* using museum archived samples in study of prospecting for zoonotic pathogens by using targeted DNA enrichment. Blue indicates museum archived samples; museum accession numbers are given (Table 1). Branches with <50% bootstrap support were collapsed. Nodal support is indicated by color coded diamonds. Scale bar indicates nucleotide substitutions per site. Assembly accession numbers (e.g., CA902374465) and tree files are available from https://doi.org/10.5281/zenodo.8014941.

**Figure 7:**
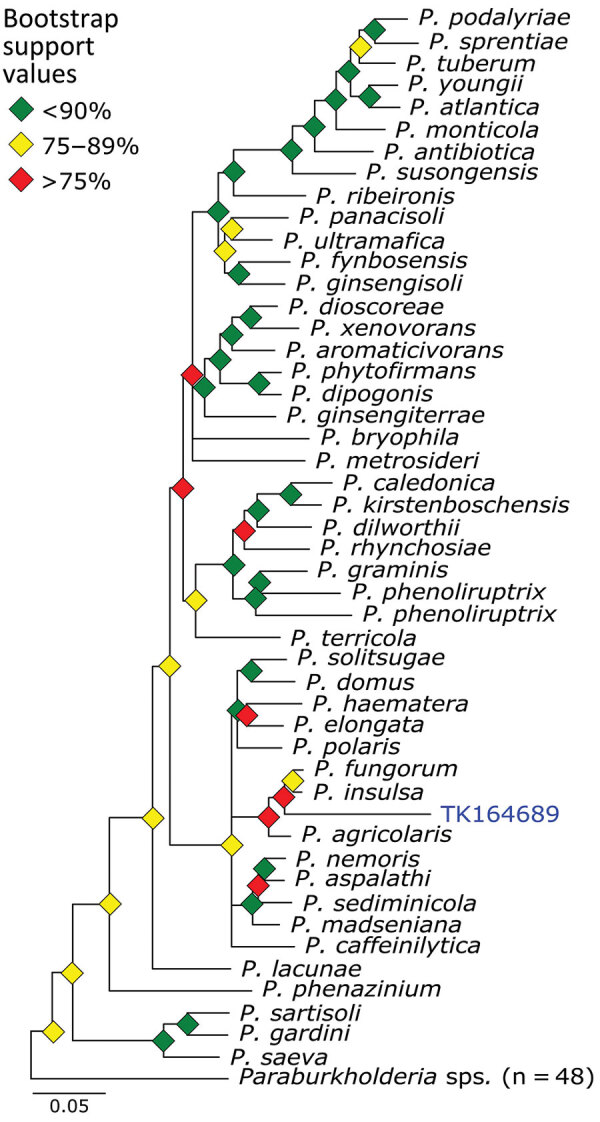
Phylogenetic analysis of *Paraburkholderia* using museum archived samples for probe panel for prospecting zoonotic pathogens by using targeted DNA enrichment. Blue indicates museum archived samples; museum accession numbers are given (Table 1). Branches with >50% bootstrap support were collapsed. Nodal support is indicated by color coded diamonds. Scale bar indicates. nucleotide substitutions per site. Assembly accession numbers (e.g., GCA90237446) and tree files are available from https://doi.org/10.5281/zenodo.8014941.

**Figure 8:**
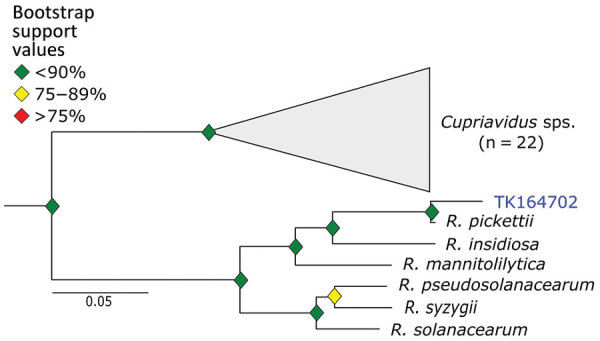
Phylogenetic analysis of *Ralstonia* using museum archived samples in study of prospecting for zoonotic pathogens by using targeted DNA enrichment. Blue indicates museum archived samples; museum accession numbers are given (Table 1). Branches with <50% bootstrap support were collapsed. Nodal support is indicated by color coded diamonds. Scale bar indicates nucleotide substitutions per site. Assembly accession numbers (e.g., GCA90237446) and tree files are available from https://doi.org/10.5281/zenodo.8014941.

We used phylogenetic analyses and rules of monophyly to identify putative pathogens to species or strain for each of the 15 genera with >1,000 reads (Figure 4, panel A). We were unable to assemble >1 target locus for any specimen in 13 genera. We were able to assemble 3–20 loci (mean = 8 loci per sample) from 16 samples containing *Bartonella* (Figure 6), 3 loci from a sample containing *Paraburkholderia* reads (Figure 7), and 8 loci from a sample containing *Ralstonia* reads (Figure 8).

We used phylogenetic analyses and rules of monophyly to identify putative pathogens to species or strain for each of the 15 genera with >1,000 reads (Figure 4, panel A). We were unable to assemble >1 target locus for any specimen in 13 genera. We were able to assemble 3–20 loci (mean 8 loci/sample) from 16 samples containing *Bartonella* (Figure 6), 3 loci from a sample containing *Paraburkholderia* reads (Figure 7), and 8 loci from a sample containing *Ralstonia* reads (Figure 8).

### Host Identification

**Figure 9:**
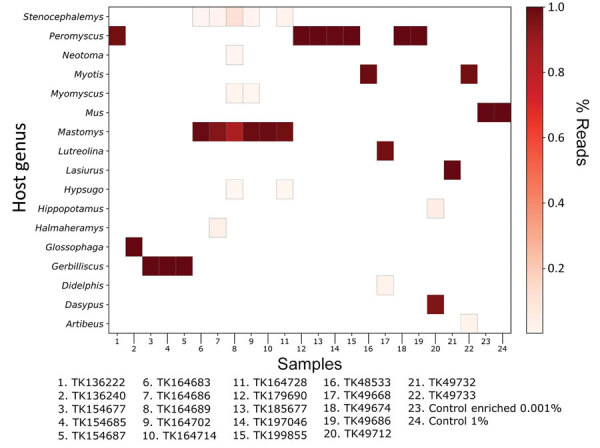
Genetic identification of mammal host from unenriched, mitochondrial reads in study of prospecting for zoonotic pathogens by using targeted DNA enrichment. Reads were compared with a database of mammalian mitochondria and assigned a taxonomic classification based on these results. A heatmap of the results shows the relative proportion of classified reads assigned to mammalian genera. Samples with <50 mitochondrial reads and single-read genera are not shown.

We compared reads from each sample to a database of mitochondrial genomes to identify the host. In general, reads from the mitochondria comprised a small proportion (<1%, mean 0.04%) of each sample (Figure 9). Despite the low number of mitochondrial reads, generic classifications from the mitochondrial database coincided with the museum identifications after filtering samples with <50 mitochondrial reads. For the remaining samples, the correct genus was identified by >85% (mean 98%) of reads from that sample. Classifying reads less than the generic level is limited by mitochondrial genome availability, but where possible, we were able to confirm museum identifications at the species level.

## Discussion

We developed a set of 39,893 biotinylated baits for targeted sequencing of >32 zoonotic pathogens, and their relatives, from host DNA samples. To test the efficacy of the bait panel, we used 4 control samples that contained either 1% or 0.001% pathogen DNA and further subdivided into pools that were enriched and unenriched. Our results (Figure 4) showed a large increase of pathogen DNA in the 1% enriched sample when compared with its unenriched counterpart. Specifically, enrichment increased the amount of pathogen DNA from 0.03% to 42.1%.

We were able to generate phylogenetically informative loci from *Plasmodium*, *Mycobacterium*, and *Schistosoma* species in the 1% enriched control sample. On the basis of genome size, we estimate genome copies as 91,611 for *Plasmodium*, 261,030 for *Mycobacterium*, and 3,159 for *Schistosoma* in the control sample. This finding indicates that the probe set is able to detect these pathogens from even a few thousand genome copies per sample (*Schistosoma* species). In contrast, we were only able to generate phylogenetically informative loci from *P. falciparum* in 0.001% enriched sample, which would hypothetically contain ≈39 genome copies. This finding implies that the bait set might be capable of identifying pathogens present in samples with only a few hundred genome copies. However, there are limitations to *Plasmodium* detection that should be considered. 

In each sample, reads were detected from only a few loci rather than from the entire genome. For example, in the 1% enriched sample, 5,879 of the 398,469 reads came from 32 loci totaling 19.6 kb. Had the unenriched sample contained the same number of reads, randomly distributed across the genome, it would have amounted to 1 read every 62 kb. We found that enrichment increased coverage at probed loci from 0.23× to 863.3×, a 3,732.3-fold increase when averaged across all pathogens/loci (Figure 4). Those results show that although large amounts of host DNA might remain in a sample, the targeted loci are greatly enriched.

We tested the panel of baits on 38, museum-archived, small mammal samples without previous knowledge of infection history. Reads from these samples were initially designated to 93 different genera, but most of these genera contained a limited number of reads. For example, almost half of the 93 genera (n = 43) were identified on the basis of a single read across all 38 samples, most likely a bioinformatic artifact. We identified 15 genera in which 1 sample had >1,000 reads. For each of these 15 genera, we extracted any reads classified within the same family (e.g., genus *Bartonella*, family Bartonellaceae) and assembled, aligned, and trimmed them for phylogenetic analyses. In most cases, the reads failed the assembly step (n = 6), were filtered on the basis of locus size or coverage (n = 5), or assembled into multiple loci that were not targeted by our bait set (n = 2); we did not pursue those reads any further. However, we were able to generate phylogenies for specimens positive for *Bartonella*, *Ralstonia*, and *Paraburkholderia* species.

*Bartonella* is a bacterial genus responsible for cat-scratch disease, Carrión’s disease, and trench fever (*34*). Transmission often occurs between humans and their pets or from infected fleas ticks, or other arthropod vectors (*35*). We were able to recover target loci for 14 of 36 specimens. A phylogeny of *Bartonella* species placed the museum samples in multiple clades (Figure 6). For example, 5 specimens formed a monophyletic clade sister to *B. mastomydis*. *B. mastomydis* recently was described from *Mastomys erythroleucus* mice collected in Senegal (*36*). Appropriately, the samples we tested were collected from *M. natalensis* mice from Botswana (Table 2). Another clade contained *B. vinsonii* and a *Sigmodon* rat (TK90542) collected in Mexico. Zoonotic transmission of *B. vinsonii* has been implicated in neurologic disorders (*37*). Other museum samples probably contain novel *Bartonella* species/strains or at least represent species/strains without genomic references.

*Paraburkholderia* is a genus of bacteria commonly associated with soil microbiomes and plant tissues. We identified *Paraburkholderia* reads in 3 specimens and were able to place 1 of those in a phylogeny sister to a clade containing *P. fungorum* and *P. insulsa*. Because bootstrap values across the phylogeny were moderate in general, and weak in this particular region (Figure 7), placement of this sample is tenuous. *P. fungorum* is the sole member of *Paraburkholderia* believed to be capable of infecting humans, but it is only a rare, opportunistic, human pathogen (*38*–*40*).

*Ralstonia* is a bacteria genus closely related to the genus *Pseudomonas*. We identified *Ralstonia* reads in 5 samples and were able to place a specimen on a phylogeny. This sample is closely affiliated with *R. pickettiii* (Figure 8). We are unaware of any examples of zoonotic transmission of *R.* *pickettii*. Rather, *R. pickettii* has been identified as a common contaminant in laboratory reagents (*41*), and outbreaks have been caused by contaminated medical supplies (*42*). We failed to identify nucleic acids in any of our negative controls during library preparation. Furthermore, if there were systemic contamination, we would expect to find *Ralstonia* species in all of our samples, rather than the 5 of 36 observed. Thus, because we cannot rule out reagent contamination, the presence of *Ralstonia* species in the museum samples should be interpreted with caution.

**Figure 10:**
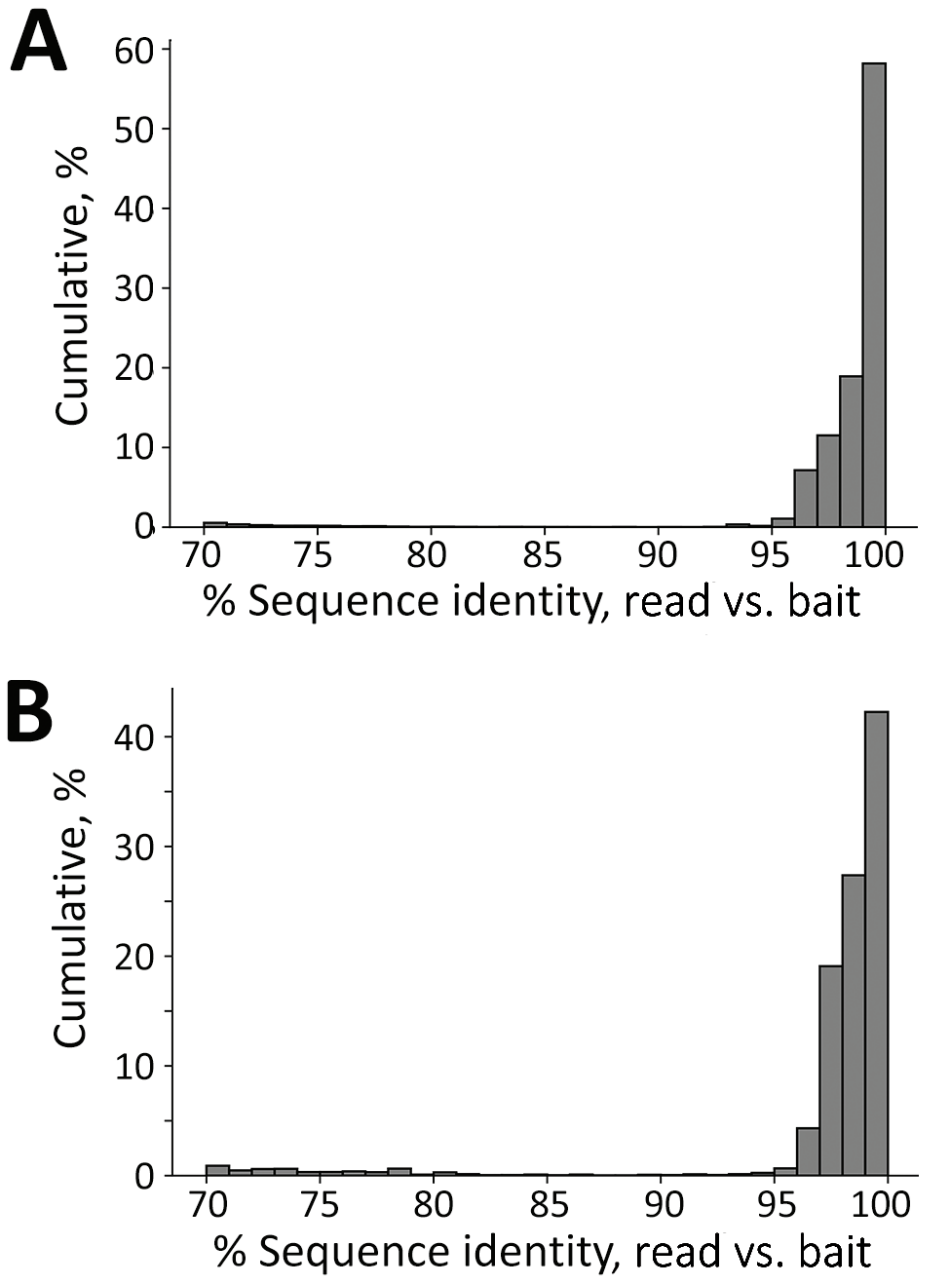
Sequence identity between enriched reads and baits in the probe panel used for targeting zoonotic pathogens in study of prospecting for zoonotic pathogens by using targeted DNA enrichment. Reads from each sample were classified against a database of target loci. Sequence identity between pathogen-derived reads and the most similar bait in the bait panel for all pathogens excluding *Bartonella* species (A) and for only *Bartonella* species (B). *Bartonella* was the most common pathogen in our samples, and the number of reads was biased toward a few individuals.

We were able to capture, sequence, and assemble loci from taxa that were not represented in the databases used to design the bait panel. This ability was possible for 2 reasons. First, the bait panel is highly redundant. The baits are sticky and able to capture nucleic acid fragments that are <10%–12% divergent (*43*). We designed the panel with <5% sequence divergence between any pair of baits at a particular locus (Figure 10). Second, sampled loci within each pathogen group spanned a range of divergences. Conserved loci were more likely to catch more divergent species that might not have been present in our initial dataset. For example, we recovered multiple species of *Bartonella* that were not present in our probe set, for which related genomes were available. However, for *Ralstonia* and *Paraburkholderia* species, we identified these samples from reads targeted by probes for the genus *Burkholderia*, a pathogenic taxon in the same family (Burkholderacea). The ability to identify taxa at these distances is because of the more conserved loci targeted by the bait panel.

During the initial read classification stage, we identified low levels of *Plasmodium* species in all but 2 museum samples, which was unexpected. Museum samples contained <3,221 *Plasmodium* reads/sample (mean 428.3 reads/sample), but we were unable to assemble them into loci for phylogenetic analyses. This limitation effectively removed those samples from downstream analyses. The *P. falciparum* genome is extremely AT rich (82%, *44*), which might result in bioinformatic false-positive results. We suspect that AT-rich, low-complexity regions of the host genome are misclassified as parasite reads. To test this hypothesis, we used fqtrim 0.9.7 (https://ccb.jhu.edu/software/fqtrim) to identify and remove low-complexity sequences within those reads. This filter by itself reduced the number of *Plasmodium* reads in the museum samples by 75.5% (maximum 298 reads, mean 57.2 reads). In comparison, only 8.2% of reads from 0.001% enriched control samples and 0.2% of reads from 1% enriched control samples were removed.

Several technical issues still need to be addressed. First, enrichment increases the targeted loci coverage by 3 orders of magnitude. However, the amount of host DNA remaining in each sample is still high. Ideally, host DNA would be rare or absent. Second, the bait panel requires relatively large up-front costs. Third, although the bait panel is developed to target a wide range of taxa, it is not possible to know which species are missed. The best way to circumvent that issue is to use controls spiked with various pathogens of interest, similar to how mock communities are used in other metagenomic studies (*45*). Those mock controls are commercially available for bacterial communities (e.g., ZymoBIOMICS Microbial Community Standards; Zymo Research, http://www.zymoresearch.com), but we have been unable to find similar products that contain eukaryotic pathogens. Solutions to those problems will make targeted sequencing with bait panels a viable tool for pathogen surveillance. Fourth, the sensitivity of the probes will depend on the sequence divergence between the probes and pathogen DNA. The more diverged the 2 are, the less efficient the capture will be. This limitation indicates that pathogen groups that have biased or limited genomic data will be less likely to capture off-target species once divergence increases by >5%–10%. Finally, the current probe panel is capable of capturing and identifying pathogens if there are >3,000 genome copies in the sample. Sensitivity needs to be improved in future iterations of the panel. One method could be to target pathogen-specific, repetitive sequences (*46*). Because those sequences are already present in the genome hundreds to thousands of times, it should be possible to greatly increase the sensitivity of the probe panel.

Although further effort is required to resolve these issues, we believe that enrichment of pathogen DNA from museum tissue samples is a viable tool worth further development. In its current form, enrichment represents a coarse tool that can be used to scan for various pathogens from archived tissues. More refined tests, such as quantitative PCR and targeted sequencing, can be used to answer taxon-specific questions. Target enrichment will be necessary for maximizing the pathogen data that are available from the hundreds of thousands of museum-archived tissues and will play a critical role in understanding our susceptibility to future zoonotic outbreaks.

Appendix 1 Additional information on prospecting for zoonotic pathogens by using targeted DNA enrichment**.**

Appendix 2 Genome information used for study on prospecting for zoonotic pathogens by using targeted DNA enrichment.
