# Supplementary material for: Prospecting for Zoonotic Pathogens by Using Targeted DNA Enrichment
Source: Emerg Infect Dis. 2023 Aug;29(8):1566–79. doi: 10.3201/eid2908.221818 (PMC10370864; doi:10.3201/eid2908.221818)
Supplement: Appendix 1 — Additional information on prospecting for zoonotic pathogens by using targeted DNA enrichment. [file 22-1818-Techapp-s1.pdf]

Article DOI: https://doi.org/10.3201/eid2908.221818

EID cannot ensure accessibility for Supplemental Materials supplied by authors. Readers who have difficulty accessing supplementary content should contact the authors for assistance.

Prospecting for Zoonotic Pathogens by Using Targeted DNA Enrichment

Appendix 1

Materials and Methods

Host-Pathogen Control Samples

We isolated DNA using the QIAGEN blood and tissue kit following manufacturer's protocol and quantified DNA using Qubit. We prepared a cocktail of pathogen DNA mixtures comprising 200 ng DNA of each pathogen (*Mycobacterium bovis*, *M. tuberculosis*, *Plasmodium vivax*, *P. falciparum*, *Schistosoma mansoni*, and *S. bovis*). A mammalian-pathogen DNA mixture was prepared by mixing pathogen DNA in DNA from uninfected liver tissues of laboratory mouse (*Mus musculus*) to make 1% and 0.001% pathogen mixtures. The negative control was prepared from the same uninfected liver tissues of laboratory mouse (*Mus musculus*) without spiking with pathogens.

Museum-Archived Samples and Controls

We extracted DNA from 42 museum samples comprising of mammalian liver tissues (in lysed buffer or frozen in liquid nitrogen) collected between 1995 and 2018 in Africa, Southern America, and the United States. Control samples were as previously described (1% and 0.001% pathogens DNA in mammalian DNA). Information for each specimen are provided in Table 2.

Computing Environment and Reproducibility

All analyses were performed on a single compute node with 48 processors and limited to 100 Gb of RAM. Bioinformatic steps were documented in a series of BASH shell scripts or Jupyter v4.9.2 notebooks. These files along with conda v4.11.0 environments are available (github.com/nealplatt/pathogen_probes) and are archived: DOI:10.5281/zenodo.7319915.

Panel Development

We developed a set of biotinylated probes for UCE-based, targeted sequencing of 32 pathogen groups (Table 1). For example, given the large evolutionary distances covered by various pathogens, we generated sets of probes that target more discrete taxonomic groups (e.g., Nemotoda, *Yersinia*). For bacterial pathogens, probes were designed to capture all species within the genus or species group. For eukaryotic pathogens, probes were designed to be effective at taxonomic ranks that ranged from species group to class. The taxonomic rank varied in eukaryotic pathogens based on the following criteria: 1) the number of available genomes, 2) sequence diversity - because this impacted the number of probes needed. Table 1 provides information on the pathogen group, targeted zoonotic agent and zoonoses.

For each group we used the Phyluce package v1.7.0 (*1*,*2*); we generated probes to target ≈49 loci using the methods described below. First, we identified orthologous loci between a focal pathogen and the remaining species in the pathogen group. Focal taxa were chosen based on their assembly contiguity or prominence as a zoonotic agent. To do this we downloaded a genome for each species in the pathogen group. Accession numbers for these assemblies are provided in Table 2. Next, we simulated 25x read coverage for each genome using the ART v2016.06.05 (*3*); read simulator with the following options: art_illumina–paired–len 100–fcov 25–mflen 200–sdev 150 -ir 0.0 -ir2 0.0 -dr 0.0 -dr2 0.0 -qs 100 -qs2 100 -na. Simulated reads from all query taxa were mapped back to a focal taxon with bbmap v38.93 (*4*); enabling up to 10% sequence divergence (minid = 0.9). Unmapped, or multimapping reads were removed using Bedtools v2.9.2 (*5*) and phyluce_probe_strip_masked_loci_from_set (filter_mask 25%). The remaining reads were merged to generate a BED file containing orthologous regions between the query and focal taxa.

Then, we identified orthologous loci among all taxa within the pathogen group using phyluce_probe_query_multi_merge_table. Next, we filtered each set of loci to retain only those shared among 33% of taxa in the pathogen group using phyluce_probe_query_multi_merge_table. We extracted 160 bp from each locus and generated an initial set of in silico probes directly from the focal genome using phyluce_probe_get_genome_sequences_from_bed and phyluce_probe_get_tiled_probes. Additional options for probe design included generating two probes per locus (-two_probes) that overlapped in the middle (-overlap-middle). Focal probes with repetitive regions or skewed GC

content (<30% or >70%) content were removed. Next, the probes from the focal taxa were mapped back to each genome in the pathogen group with phyluce_probe_run_multiple_lastzs_sqlite. We used the –identity option to limit searches with a maximum divergence of 30%. Using these results, we extracted 120-bp loci from the probed regions in each representative genome extracted using phyluce_probe_slice_sequence_from_genomes. Theoretically, this dataset should contain orthologous 120-bp sequences from most taxa in each pathogen group. We verified this with phyluce_probe_get_multi_fasta_table, which provides a table showing the number of taxa identified at each locus. We used this information to identify the 100 loci capable of capturing most taxa from the pathogen group. Next, we generated two 80-bp probes from each of the 100-bp and 120-bp loci. We used phyluce_probe_easy_lastz to compare the probes to themselves and remove any that were possible duplicates. Then we reduced the probe set even further by clustering probes based on sequence identity with cd-hit-est v4.8.1 (*6*). We identified sequence clusters with >95% similarity and retained only 1 probe per group. Finally, we recalculated the number of probes needed to capture each locus.

The proceeding steps were repeated for each pathogen group shown in Table 1. To generate a final panel, we selected 49 loci per pathogen group in a way that minimized the number of probes needed. In some cases, we needed to generate 2 sets of probes to adequately represent target pathogens. For example, Kinetoplastea contains 2 pathogens of interest, *Trypanosoma* and *Leishmania*. The baits designed for *Leishmania* were able to target all 49 loci in the most of the Kinetoplastea but only 23 loci in *Trypanosoma*. We then generated a second set of 617 *Trypanosoma*-specific baits to augment the kinetoplastid baits and ensure that *Trypanosoma* parasites were represented adequately in the final panel. Likewise, we doubled the number of baits used to capture loci from the *Bacillus cereus* group to effective capture *B. cereus* and *B. anthracis*. The probe set was quality checked by Arbor Bioscienes. This included comparing the probe set to mammal genomes with blastn v2.12.0 (*7*) and checking for low-complexity sequences. Any probes that failed quality control were replaced before synthesis.

Library Preparation

Standard DNA sequencing libraries were generated from 500 ng of DNA per sample. We used the KAPA Hyperplus kit protocol with the following modifications: 1) enzymatic fragmentation at 37°C for 10 minutes, 2) adaptor ligation at 20°C for an hour, and 3) four cycles

of library PCR amplification. To minimize adaptor switching we used unique dual indexed (UDI) adapters (IDT xGen Stubby Adaptor-UDI Primers). Each library was eluted in 20 µL of sterile water and the base pairs sizes and concentration estimated by Agilent 4200 Tapestation (Figure 2).

Individual samples with similar DNA concentrations were combined together into pools of 4–16 samples and the total volume was reduced to 7 µL with a speedvac vacuum concentrator. Next, we used the high sensitivity protocol of myBaits v.5 (Daicel Arbor Biosciences) to enrich target pathogen loci from the host/pathogen control and museum archived samples. We used 2 rounds of enrichment for each pool of samples. Probe concentration was 100 ng/µL. Each round was 24 hours at 65°C. After washing of unbound DNA, each library was amplified with a 15-cycle PCR amplification step and quantified using qPCR. Finally, the pools of 4–16 were combined into an equimolar pool for sequencing. All sequencing reactions were on single lanes of Illumina Hi-Seq 2500.

Bioinformatic Analyses

All analyses were performed on a single compute node with 48 processors and limited to 100 Gb of RAM. Bioinformatic steps were documented in a series of BASH shell scripts or Jupyter notebooks. These files along with conda environments are available at github.com/nealplatt/pathogen_probes and archived. The basic structure of the bioinformatic analyses are shown in Figure 3. In general, we used the Kraken2 v2.1.2 (*8*) to assign a taxonomic id to each read, the Phyluce v1.7.1 (*1*,*2*) pipeline to identify, assemble, and align loci, and RaxML-NG v1.0.1 to generate phylogenies from each pathogen group of interest.

First, we used Trimmomatic v0.39 (*9*) to trim and quality filter low-quality bases and Illumina adapters. Then, we used Kraken2 v2.1.1 (*8*) to compare each read from our samples to a reduced dataset of target loci using a –conf cutoff of 0.2. We decided to compare our reads to a reduced dataset of target loci to minimize the computational expense of these comparison. To generate the reduced database of bait-targeted loci, we downloaded one representative or reference genome from all species in RefSeq v212 (*10*) with genome_updater.sh v0.5.1 (https://github.com/pirovc/genome_updater). Then we used BBMap v38.96 (*4*) to map all the baits to each genome and a kept the 10 best sites that mapped with ≥85% sequence identity.

Next, we extracted these hits along with 1,000 bp up and downstream. These sequences were combined into a single fasta file that should contain the major mapping locations for our baits.

Once reads were classified we identified genera that were known pathogens or were present in at least one sample with more than 1,000 reads. Next, we extracted reads from the relevant family with KrakenTools v1.2 (https://github.com/jenniferlu717/KrakenTools/). These reads were then assembled (Figure 3, panel B) with the SPAdes genome assembler v3.14.1 (*11*) using the phyluce_assembly_assemblo_spades wrapper script. We filtered out low quality contigs based on size (<100 bp) and median coverage (<10×) as calculated by the SPAdes genome assembler. Next, we filtered individuals even further by removing individuals with fewer <2 contigs.

While we were assembling and filtering contigs from each isolated target loci from species with available genome assemblies, we used genome_updater.sh v0.5.1 (https://github.com/pirovc/genome_updater) to download one (-A 1) reference or representative (-c reference genome, representative genome) genome from either refseq or Genbank (-d refseq,genbank) for the pathogen group. We also included at least 1 individual from an outlier genus to root downstream analyses. These genomes were converted to twobit format with faToTwoBit. Next, we used phyluce_probe_run_multiple_lastzs_sqlite to compare probes from the pathogen group to the genome assemblies with an identity cut off of 85% (–identity 0.85). These loci plus 1 kb of flanking sequence (–flank 1000) were extracted from the genome using phyluce_probe_slice_sequence_from_genomes. After extraction, the sliced loci were identified and counted using phyluce_assembly_match_contigs_to_probes (–min-identity 90) and phyluce_assembly_get_match_counts. Next, we combined the loci generated from our samples with those from representative and reference genomes and aligned them with phyluce_align_seqcap_align. The resulting alignments were trimmed with gblocks v0.91b (*12*) and phyluce_align_get_gblocks_trimmed_alignments_from_untrimmed. We then counted the number of taxa per locus alignment (phyluce_align_get_taxon_locus_counts_in_alignments) and removed taxa with fewer than 2 loci (phyluce_align_extract_taxa_from_alignments). Then we removed any loci that contain fewer than half of the expected number of taxa with phyluce_align_get_only_loci_with_min_taxa and concatenated the remaining loci into a single phylip alignment (phyluce_align_concatenate_alignments).

We used RaxML-NG v1.0.1 (*13*) to generate a maximum-likelihood phylogenetic tree from the concatenated alignment. We ran 100 parsimony tree searches and then another 1,000 replicates using the GTR + G substitution model. Branches with less than 50% support were collapsed with the Newick Utilities v1.6 (*14*), Newick editor (nw_ed <input_tree_file>'i and b< = 50'). These steps were then repeated with other pathogen groups identified in the samples.

Host Identification

We verified museum identifications by comparing reads to a second Kraken2 v2.1.2 (*8*) database containing mammalian mitochondrial genomes. To do this, we downloaded all available mammalian mitochondrial genomes (n = 1,651) from https://www.ncbi.nlm.nih.gov/genome/organelle/ (last accessed 3 November 2022). We then created a custom database and compared each of our samples sing Kraken2 and no confidence cutoffs. The Kraken2 classifications were filtered by removing any samples with fewer than 50 classified reads and any single-read, generic classifications.

References

1. Faircloth BC, McCormack JE, Crawford NG, Harvey MG, Brumfield RT, Glenn TC. Ultraconserved elements anchor thousands of genetic markers spanning multiple evolutionary timescales. Syst Biol. 2012;61:717–26. PubMed https://doi.org/10.1093/sysbio/sys004

2. Faircloth BC. Identifying conserved genomic elements and designing universal bait sets to enrich them. Methods Ecol Evol. 2017;8:1103–12. https://doi.org/10.1111/2041-210X.12754

3. Huang W, Li L, Myers JR, Marth GT. ART: a next-generation sequencing read simulator. Bioinformatics. 2012;28:593–4. PubMed https://doi.org/10.1093/bioinformatics/btr708

4. Bushnell B. BBMap: a fast, accurate, splice-aware aligner. Berkeley (CA): Lawrence Berkeley National Laboratory; 2014.

5. Quinlan AR, Hall IM. BEDTools: a flexible suite of utilities for comparing genomic features. Bioinformatics. 2010;26:841–2. PubMed https://doi.org/10.1093/bioinformatics/btq033

6. Fu L, Niu B, Zhu Z, Wu S, Li W. CD-HIT: accelerated for clustering the next-generation sequencing data. Bioinformatics. 2012;28:3150–2. PubMed https://doi.org/10.1093/bioinformatics/bts565

7. Altschul SF, Gish W, Miller W, Myers EW, Lipman DJ. Basic local alignment search tool. J Mol Biol. 1990;215:403–10. PubMed https://doi.org/10.1016/S0022-2836(05)80360-2

8. Wood DE, Lu J, Langmead B. Improved metagenomic analysis with Kraken 2. Genome Biol. 2019;20:257. PubMed https://doi.org/10.1186/s13059-019-1891-0

9. Bolger AM, Lohse M, Usadel B. Trimmomatic: a flexible trimmer for Illumina sequence data. Bioinformatics. 2014;30:2114–20. PubMed https://doi.org/10.1093/bioinformatics/btu170

10. O'Leary NA, Wright MW, Brister JR, Ciufo S, Haddad D, McVeigh R, et al. Reference sequence (RefSeq) database at NCBI: current status, taxonomic expansion, and functional annotation. Nucleic Acids Res. 2016;44(D1):D733–45. PubMed https://doi.org/10.1093/nar/gkv1189

11. Bankevich A, Nurk S, Antipov D, Gurevich AA, Dvorkin M, Kulikov AS, et al. SPAdes: a new genome assembly algorithm and its applications to single-cell sequencing. J Comput Biol. 2012;19:455–77. PubMed https://doi.org/10.1089/cmb.2012.0021

12. Talavera G, Castresana J. Improvement of phylogenies after removing divergent and ambiguously aligned blocks from protein sequence alignments. Syst Biol. 2007;56:564–77. PubMed https://doi.org/10.1080/10635150701472164

13. Kozlov AM, Darriba D, Flouri T, Morel B, Stamatakis A. RAxML-NG: a fast, scalable and user-friendly tool for maximum likelihood phylogenetic inference. Bioinformatics. 2019;35:4453–5. PubMed https://doi.org/10.1093/bioinformatics/btz305

14. Junier T, Zdobnov EM. The Newick utilities: high-throughput phylogenetic tree processing in the UNIX shell. Bioinformatics. 2010;26:1669–70. PubMed https://doi.org/10.1093/bioinformatics/btq243

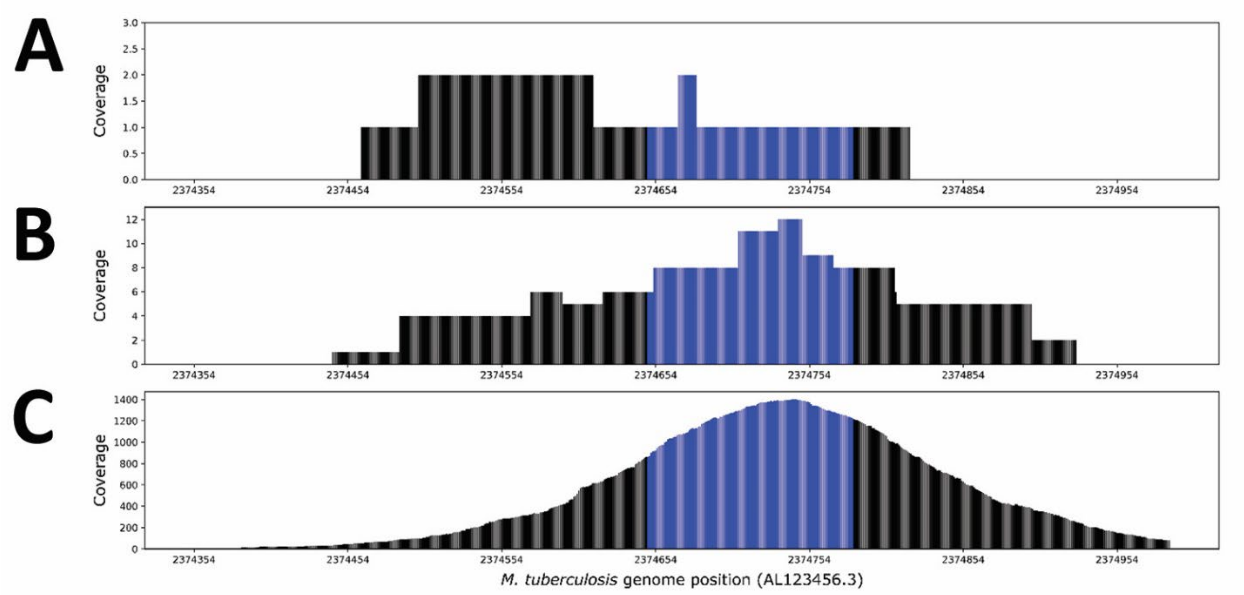

Appendix Figure. Read depth at a targeted region in *Mycobacterium tuberculosis* in the A) 1%, unenriched, B) 0.001% enriched, and C) 1% enriched control, samples. This particular probe was designed for (AL123456.3:2,374,648–2,374,781; shown in blue). Median coverage at this locus increased from 1x in the 1% unenriched sample (A) to 8x in the 0.001% enriched sample (B) and 1,278x in the 1% enriched control sample (C).
